# Association of Body Mass Index with Timing of Death during Tuberculosis Treatment

**DOI:** 10.1371/journal.pone.0170104

**Published:** 2017-01-13

**Authors:** Hsin-Hao Lai, Yun-Ju Lai, Yung-Feng Yen

**Affiliations:** 1 Section of Infectious Diseases, Taipei City Hospital, Taipei City Government, Taipei, Taiwan; 2 Division of Endocrinology and Metabolism, Department of Internal Medicine, Puli Branch of Taichung Veterans General Hospital, Nantou, Taiwan; 3 School of Medicine, National Yang-Ming University, Taipei, Taiwan; Johns Hopkins University Bloomberg School of Public Health, UNITED STATES

## Abstract

**Background:**

The association between body mass index and mortality in patients with tuberculosis has not been extensively studied, and the existing evidence is inconsistent. This study aimed to investigate the impact of body mass index on timing of death in patients with tuberculosis.

**Methods:**

All Taiwanese adults with tuberculosis in Taipei, Taiwan, were included in a retrospective cohort study in 2011–2012. Multinomial logistic regression was used to evaluate the association between body mass index and timing of death in patients with tuberculosis.

**Results:**

Among 1557 eligible patients, 84.1% (1310), 8.2% (128), and 7.6% (119) underwent successful treatment, early death, and late death, respectively. The mean age of the patients with tuberculosis was 64.2 years old, and 67.7% were male. After controlling for potential confounding variables, underweight with body mass index less than 18.5 kg/ m^2^ was significantly associated with elevated risk of all-cause mortality [Adjusted odds ratio (AOR), 1.64; 95% confidence interval (CI), 1.17–2.30]. Considering timing of death, underweight with body mass index less than 18.5 was significantly associated only with elevated risk of early mortality within the first 8 weeks of treatment onset (AOR, 2.22; 95% CI, 1.45–3.40)

**Conclusions:**

For patients with tuberculosis infection, underweight with body mass index less than 18.5 kg/ m^2^ is an independent predictor for early mortality within the first 8 weeks of treatment.

## Introduction

Tuberculosis (TB) remains a common and deadly disease globally. Even though the incidence rate of TB infection has decreased since 2001, when the Millennium Development Goals were instituted, mortality caused by TB cases is still high [1].

In Taiwan, TB has been the most prevalent reported infectious disease for decades [2]. In 2006, Taiwan’s Centers for Disease Control (CDC) adopted a directly observed therapy, short course (DOTS) program intended to achieve a successful treatment rate of 85% and halve TB incidence by 2015. Since then, the success rate for TB treatment improved slightly to 72.7% in 2011. However, mortality among patients with TB remains high, and 19.6% of new TB cases in 2011 resulted in death [2].

The vicious cycle of malnutrition and infection, whereby malnutrition increases the risk of infection and vice versa, has been discussed in the past [3]. Body mass index (BMI) is a popular and useful tool to evaluate nutrition status, and lower BMI is strongly associated with higher mortality, whether overall or cause-specific (including TB-infection-related [4]. Some reports also indicate higher mortality among patients with TB who have lower BMI [5–11]. However, the relationship between BMI and timing of death during TB treatment has not been well established.

Although few studies have examined prognostic factors of TB mortality regarding the timing of death [12], a recent report showed that predictors of mortality varied according to different timing of death in patients with TB [13]. To our best knowledge, almost no studies have examined whether the impact of BMI on mortality varied according to different timing of death

Development of effective interventions to improve TB outcomes requires better understanding of the factors associated with timing of death in patients with TB. This population-based study thus aimed to investigate the impact of BMI on mortality with respect to timing of death in patients with TB.

## Materials and Methods

### Study population and data source

This retrospective cohort study employed TB surveillance data from Taipei, Taiwan. In Taipei, there is an obligation to report TB cases to the Taipei TB Prevention Center within seven days of diagnosis. This study included Taiwanese adults (age ≥18 years) diagnosed with TB in Taipei in 2011–2012. TB was defined by positive clinical and/or laboratory findings [14]. This project was approved by the Institutional Review Board of Taipei City Hospitals and the data were analyzed anonymously. The patients’ written consents were waived by the approving IRB because personally identifying information were not included in the dataset.

### Data collection

When patients with TB were reported to the Taipei TB Prevention Center, trained case managers used a structured questionnaire to interview patients about their sociodemographic characteristics, clinical findings, and comorbid diseases. The sociodemographic factors included age, gender, BMI, marital status, education level, smoking status and employment. Patients with TB in Taipei are required by law to be monitored until treatment success, death, or loss to follow-up. To monitor treatment response, case managers followed up with all patients with TB by phone or in person biweekly.

### Outcome variable

The outcome variable of interest was treatment outcome, which was categorized into two groups: successful treatment with no death and mortality. Mortality was classified as early or late death according to timing of death. Early death was defined as occurring within the first 8 weeks of TB treatment, and late death was defined as mortality later than 8 weeks after the start of TB treatment [15]. The successful treatment group was used as the reference.

### Main explanatory variable

The main explanatory variable was body mass index (BMI, defined as kg/m^2^). BMI was recorded when case managers interviewed patients with TB at the time of notification to the Taipei TB Prevention Center. In accordance with the WHO International Classification of Adult Body Weight, BMI was categorized as underweight (<18.5 kg/m^2^), normal (18.5–24.9 kg/m^2^), or overweight (≥25 kg/m^2^) [16].

### Control variables

Covariates identified in previous studies as risk factors for TB mortality were assessed in the analyses, including subject sociodemographic factors (e.g., education level, smoking status), clinical findings [i.e., acid-fast bacilli (AFB) smear status, TB culture, cavities on chest radiograph, pleural effusion, extrapulmonary TB], and comorbidities (e.g., malignancy, diabetes mellitus, end-stage renal disease). Education level was separated into categories of uneducated, elementary school, high school, and university or higher. Smoking status was categorized as never smoking, quit smoking, or currently smoking.

### Statistical analysis

First, the study subjects’ demographic characteristics were analyzed. Continuous data were described as mean (SD), and two-sample t tests were used for comparisons between groups. Categorical data were analyzed by Pearson χ^2^ tests, where appropriate.

Multivariate analyses were conducted using death versus no death as the outcome, with BMI category as the main explanatory variable. A multinomial regression model was used to identify factors associated with timing of death (i.e., early versus late death). We also tested the interaction between BMI and other covariates in the multivariate analysis. Adjusted odds ratios (AORs) with 95% confidence intervals (CIs) are reported to indicate the associations’ strength and direction.

To examine the gender difference on the association of BMI and mortality, this study analyze the data after stratifying study subjects by gender. All data management and analyses were performed using the SAS 9.4 software package (SAS Institute, Cary, NC).

## Results

### Characteristics of patients with TB

In total, 1869 TB cases were reported to the Taipei TB Prevention Center in 2011–2012. Of those patients, 46, 20, 227, and 19 died before starting TB treatment, were lost to follow-up during treatment, had incomplete data, and were still receiving treatment at the time of this study, respectively. The remaining 1557 were included in subsequent analyses. Overall, the mean age of included subjects was 64.2 years old (range 18–112 years old), and 67.7% were male. According to the WHO definition, the BMIs of 25.0%, 63.6%, and 11.4% of subjects were classified as underweight, normal, and overweight, respectively. During the study follow-up period, 137 (13.8%), 91 (23.4%), and 19 (10.7%) deaths occurred in normal-weight, underweight, and overweight patients, respectively.

Table 1 shows the baseline characteristics of TB patients, by treatment outcome. As compared with TB patients with treatment success, patients with early or late death were older, higher proportion of comorbidities [e.g., end-stage renal disease (ESRD) and malignancy], lower educational level and more unemployment. Besides, patients with early or late death had higher rate of AFB and TB culture positivity than those with treatment success.

**Table 1:** Baseline Characteristics of Tuberculosis patients, by treatment outcome.

Characteristics		Treatment outcome			P value
		Treatment success (n = 1310) %	Early death in TB treatment (n = 128) %	Late death in TB treatment (n = 119) %	
Body mass index (kg/m^2^)					
	Normal (18.5–24.9)	65.1	47.7	63.9	<.001
	Underweight (<18.5)	22.8	44.5	28.6	
	Overweight (≥25)	12.1	7.8	7.5	
Age (years)					
	Mean ± SD	60.9 ± 20.0	82.9 ± 10.5	79.9 ± 12.0	<.001
	18–64	52.7	7.8	10.9	<.001
	≥65	47.3	92.2	89.1	
Gender					
	Female	34.2	21.9	22.7	0.001
	Male	65.8	78.1	77.3	
Marital status					
	Unmarried	22	5.5	7.6	<.001
	Married	78	94.5	92.4	
Education level					
	No education	7.9	15.6	11.8	<.001
	Elementary school	20	33.6	39.5	
	High school	37.6	34.4	31.1	
	University or higher	34.5	16.4	17.6	
Unemployment					
	No	28.8	3.9	5.9	<.001
	Yes	71.2	96.1	94.1	
Smoking status					
	Never smoking	78.4	79.6	83.2	0.026
	Quit smoking	5.7	10.2	8.4	
	Current smoking	15.9	10.2	8.4	
DM					
	No	83.2	83.6	83.2	0.994
	Yes	16.8	16.4	16.8	
ESRD					
	No	97.3	94.5	87.4	<.001
	Yes	2.7	5.5	12.6	
Malignancy					
	No	93.7	78.9	75.6	<.001
	Yes	6.3	21.1	24.4	
History of TB disease					
	No	95.4	94.5	95	0.886
	Yes	4.6	5.5	5	
AFB-smear positivity					
	No	62.8	36.7	60.5	<.001
	Yes	37.2	63.3	39.5	
TB-culture positivity					
	No	28.7	13.3	22.7	<.001
	Yes	71.3	86.7	77.3	
Cavity on CXR					
	No	85.4	86.7	92.4	0.104
	Yes	14.6	13.3	7.6	
Pleural effusion					
	No	89.5	78.9	84	<.001
	Yes	10.5	21.1	16	
Extrapulmonary TB					
	No	91.5	98.4	94.1	0.015
	Yes	8.5	1.7	5.9	

DM, diabetes mellitus; ESRD, end-stage renal disease; TB, tuberculosis; AFB, acid-fast bacilli; CXR, chest radiograph.

### Univariate analysis of the protective and risk factors for all-cause mortality

As shown in Table 2, the univariate analysis revealed that factors associated with high risk of all-cause death included underweight, age ≥65 years, male gender, being married, unemployment, ESRD, malignancy, positive AFB smear, positive TB culture, and pleural effusion on chest radiograph (CXR). Also, variables associated with lower risk of all-cause death included high school, university or higher education, current smoking, and extrapulmonary TB.

**Table 2:** Univariate and Multivariate Analyses of Risk Factors for All-cause Mortality in Patients with TB, Taipei, Taiwan (2011–2012).

Factor		Number of patients	Deaths	Univariate analysis	Multivariate analysis
			n (%)	OR (95% CI)	AOR (95% CI)
Body mass index (kg/m^2^)					
	Normal (18.5–24.9)	990	137 (13.8)	1	1
	Underweight (<18.5)	389	91 (23.4)	1.90 (1.41–2.56)***	1.64 (1.17–2.30)**
	Overweight (≥25)	178	19 (10.7)	0.74 (0.45–1.24)	0.84 (0.48–1.46)
Age (years)					
	18–64	713	23 (3.2)	1	1
	≥65	844	224 (26.5)	10.84 (6.96–16.87)***	5.36 (3.16–9.10)***
Sex					
	Female	503	55 (10.9)	1	1
	Male	1054	192 (18.2)	1.81 (1.32–2.50)***	1.60 (1.09–2.33)*
Marital status					
	Unmarried	304	16 (5.3)	1	1
	Married	1253	231 (18.4)	4.07 (2.41–6.87)***	1.20 (0.64–2.26)
Education level					
	No education	138	34 (24.6)	1	1
	Elementary school	352	90 (25.6)	1.05 (0.67–1.66)	0.96 (0.58–1.58)
	High school	573	81 (14.1)	0.50 (0.32–0.79)**	0.67 (0.40–1.12)
	University or higher	494	42 (8.5)	0.28 (0.17–0.47)***	0.51 (0.29–0.91)*
Unemployment					
	No	389	12 (3.1)	1	1
	Yes	1168	235 (20.1)	7.91 (4.38–14.31)***	2.27 (1.18–4.36)*
Smoking status					
	Never smoking	1228	201 (16.4)	1	1
	Quit smoking	98	23 (23.5)	1.57 (0.96–2.56)	1.12 (0.65–1.95)
	Current smoking	231	23 (10.0)	0.57 (0.36–0.89)*	0.79 (0.46–1.95)
DM					
	No	1296	206 (15.9)	1	1
	Yes	261	41 (15.7)	0.99 (0.68–1.42)	0.94 (0.63–1.41)
ESRD					
	No	1500	225 (15.0)	1	1
	Yes	57	22 (38.6)	3.56 (2.05–6.18)***	2.80 (1.49–5.25)**
Malignancy					
	No	1419	191 (13.5)	1	1
	Yes	138	56 (40.6)	4.39 (3.03–6.37)***	3.90 (2.55–5.97)***
TB relapse					
	No	1484	234 (15.8)	1	1
	Yes	73	13 (17.1)	1.16 (0.63–2.14)	0.94 (0.47–1.89)
AFB smear					
	Negative	942	119 (12.6)	1	1
	Positive	615	128 (20.8)	1.82 (1.38–2.39)***	1.87 (1.33–2.64)***
TB culture					
	Negative	420	44 (10.5)	1	1
	Positive	1137	203 (17.9)	1.86 (1.31–2.63)***	1.17 (0.75–1.82)
Cavities on CXR					
	No	1340	221 (16.5)	1	1
	Yes	217	26 (12.0)	0.69 (0.45–1.06)	0.83 (0.50–1.39)
Pleural effusion on CXR					
	No	1347	201 (14.6)	1	1
	Yes	183	46 (25.1)	1.96 (1.36–2.83)***	1.95 (1.27–3.00)**
Extrapulmonary TB					
	No	1437	238 (16.6)	1	1
	Yes	120	9 (7.5)	0.41 (0.20–0.82)**	0.68 (0.31–1.49)

* <.05;

** <.01;

*** <.001;

DM, diabetes mellitus; ESRD, end-stage renal disease; TB, tuberculosis; AFB, acid-fast bacilli; CXR, chest radiograph; AOR, adjusted odds ratio; CI, confidence interval.

### Association between BMI and all-cause mortality

Multivariate logistic regression analysis was used to identify independent risk factors for all-cause mortality in patients with TB (Table 2). After adjusting for socio-demographic characteristics, clinical findings, and comorbidities, the risk of all-cause mortality was significantly higher in underweight patients (AOR, 1.64; 95% CI, 1.17–2.30; *P* = .002) than in normal-weight patients. Overweight was not significantly associated with all-cause mortality in patients with TB. Other risk factors associated with all-cause mortality included age ≥65 years (AOR, 5.36; 95% CI, 3.16–9.10), male gender (AOR, 1.60; 95% CI, 1.09–2.33), unemployment (AOR, 2.27; 95% CI, 1.18–4.36), ESRD (AOR, 2.80; 95% CI, 1.49–5.25), malignancy (AOR, 3.90; 95% CI, 2.55–5.97), positive AFB smear (AOR, 1.87; 95% CI, 1.33–2.64), and pleural effusion on CXR (AOR, 1.95; 95% CI, 1.27–3.00). Factors associated with lower risk of all-cause mortality included education level of university or higher (AOR, 0.51; 95% CI, 0.29–0.91). The interaction terms between BMI and other covariates were not statistically significant in the multivariate analysis.

### Association between BMI and timing of death during TB treatment

Multinomial regression showed that, as comparing to no death, underweight BMI was significantly associated with elevated risk of early death (AOR, 2.22; 95% CI, 1.45–3.40; *P* < .001) but not significantly associated with late death during TB treatment (Table 3). Moreover, overweight BMI was not significantly associated with early or late death during TB treatment.

**Table 3:** Multinomial regression for association between BMI and timing of death in Patients with TB^a^.

		Early death in TB treatment	Late death in TB treatment
		AOR (95% CI)	AOR (95% CI)
Body mass index (normal weight)			
	Underweight	2.22 (1.45–3.40)***	1.14 (0.72–1.81)
	Overweight	0.94 (0.45–1.97)	0.76 (0.36–1.60)
Age ≥65 years		6.43 (3.04–13.63)***	4.65 (2.31–9.36)***
Male gender		1.51 (0.91–2.49)	1.68 (1.02–2.78)*
Married status		1.48 (0.61–3.57)	0.99 (0.44–2.19)
Education level			
	No education	1	1
	Elementary school	0.81 (0.43–1.52)	1.16 (0.59–2.29)
	High school	0.64 (0.34–1.21)	0.72 (0.36–1.45)
	University or higher	0.46 (0.22–0.95)*	0.59 (0.27–1.28)
Smoking status			
	Never smoking	1	1
	Quit smoking	1.33 (0.66–2.66)	0.94 (0.44–1.98)
	Current smoking	0.91 (0.46–1.81)	0.69 (0.33–1.43)
Unemployment		2.74 (1.03–7.23)*	1.94 (0.84–4.50)
DM		0.98 (0.58–1.68)	0.89 (0.52–1.54)
ESRD		1.72 (0.69–4.30)	3.83 (1.90–7.73)***
Malignancy		3.75 (2.18–6.43)***	4.10 (2.45–6.89)***
History of TB disease		0.92 (0.38–2.23)	0.98 (0.39–2.50)
Positive AFB smear		2.83 (1.79–4.46)***	1.21 (0.77–1.91)
Positive TB culture		1.30 (0.68–2.47)	1.09 (0.63–1.89)
Cavity on CXR		0.96 (0.52–1.77)	0.67 (0.32–1.44)
Pleural effusion		2.76 (1.61–4.74)***	1.38 (0.77–2.46)
Extrapulmonary TB		0.37 (0.08–1.63)	0.85 (0.35–2.06)

^a^Reference: successfully treated individuals.

* <.05;

** <.01;

*** <.001;

AOR = adjusted odds ratio; CI = confidence interval; DM, diabetes mellitus; ESRD, end-stage renal disease; TB, tuberculosis; AFB, acid-fast bacilli; CXR, chest radiograph.

Other factors associated with early and late death included age ≥65 years and malignancy. Risk factors for early death included unemployment, positive AFB smear, and pleural effusion on CXR, and university or higher education was associated with reduced risk of early death. Furthermore, male gender and ESRD were associated with elevated risk of late death.

### Association between BMI and mortality in male and female patients

**Fig 1:**
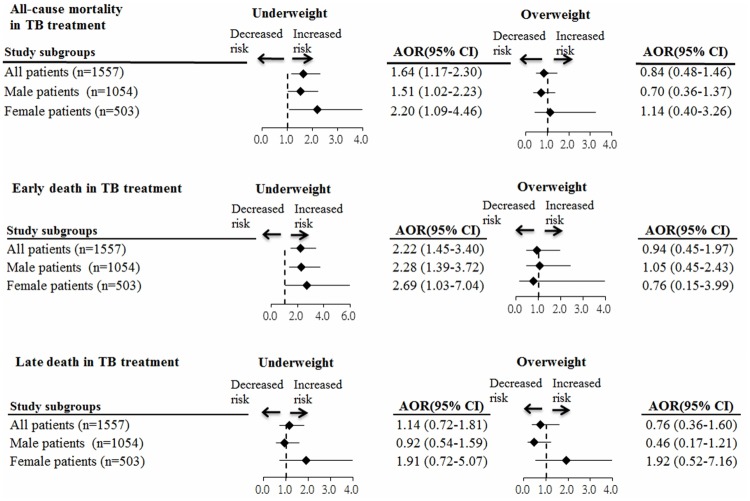
Subgroup analysis of the association between BMI and mortality after stratifying patients by gender. Values greater than 1.0 indicate increased risk. AOR = adjusted odds ratio, TB = tuberculosis.

Fig 1 shows the results of analysis of the association between BMI and mortality after stratifying patients by sex. Underweight was significantly associated with higher risks of all-cause and early death, but not significantly associated with late death during treatment in male and female patients. Moreover, overweight BMI was not significantly associated with all-cause, early, or late death during TB treatment in male and female patients.

## Discussion

A total of 1557 Taiwanese adults with TB infection were enrolled in this cohort study in 2011–2012, and their overall mortality rate was 15.9%. Underweight was significantly associated with elevated risk of all-cause mortality during treatment after controlling for potential confounding variables, while overweight was not. When timing of death was considered, underweight was only significantly associated with elevated mortality risk within the first 8 weeks of treatment, not later than 8 weeks after treatment onset.

We identified individual socioeconomic status variables that were risk factors for mortality. Specifically, individuals with education of university or higher had lower risk of all-cause and early death during the TB treatment. However, unemployment was significantly associated with a higher risk of all-cause and early death during the TB treatment. Education level and job status are indices of individual socioeconomic status [17]. Previous studies have found that individuals with higher socioeconomic status have greater awareness and knowledge of TB [18], and are less likely to have delay in the diagnosis and treatment of TB [19]. Furthermore, prior reports showed that TB patients with higher socioeconomic status are more likely to have good adherence to TB treatment [20, 21], which could reduce the mortality during TB treatment. To improve TB treatment outcomes, future control programmes should target particularly patients who had a lower socioeconomic status.

The association between malnutrition and timing of death in patients with TB during treatment has not been well-researched. Previous studies using BMI [5–11], body weight [22–24] or serum albumin [25–27] as markers to evaluate nutritional status have indicated that malnutrition could cause elevated mortality during treatment in patients with TB. However, few studies have mentioned the timing of mortality in patients with TB who are under treatment. Zachariah et al [11] found that patients with TB and BMI < 17.0 kg/m^2^ had elevated mortality risk within 4 weeks of treatment. A retrospective study conducted in Lima, Peru, showed that patients with multidrug-resistant TB and low BMI had increased mortality within 4 months after treatment onset [7]. Low body weight has also been connected with elevated early mortality risk in three other studies, but the definition of early mortality was not clear in those reports [22–24]. Sacks et al. documented low body weight as a clinical predictor for early death; Santha et al. mentioned that early death was independently associated with body weight less than 35 kg; and Elliott et al. showed that low body weight was associated with elevated levels of death within the first month of treatment among patients with human immunodeficiency virus (HIV) and TB coinfection. Moreover, low serum albumin has been indicated as an independent predictor of death within 1 month in patients with TB being treated in an ICU [27]. The present study indicated that patients with TB and underweight BMI < 18.5 kg/m^2^ had higher early mortality within the first 8 weeks of treatment, while normal and overweight patients did not. The evidence was strengthened by using multinomial regression to evaluate the association between BMI and timing of death in patients with TB under treatment: underweight was only associated with elevated risk of death within the first 8 weeks of treatment, not after 8 weeks.

The reasons that underweight is a significant predictor of early death might extend beyond the worsened severity of lung infections in low-BMI patients with TB noted in clinical cases [28]. Such patients’ immune systems might also lose control of Mycobacteria tuberculosi*s* because of suppression of T cells caused by decreasing lymphocyte stimulation, Th1 cytokines, and elevated production of transforming growth factor β, as noted in animal experiments [3].

Several limitations should be acknowledged when interpreting the findings of this single-city, population-based study. First, 25.9% of TB cases were diagnosed by clinical presentation rather than microbiological evidence, such as AFB smear or culture, which might have caused overdiagnosis of TB infection. Despite this might have resulted in overdiagnosis of TB, such overdiagnosis is unlikely to be widespread because an expert committee is held monthly by the Taipei TB Control Department to discuss uncertain TB case diagnoses [2]. Second, since this was a retrospective cohort study, some important information on the patients with TB (e.g., income status, whether or not they used intravenous drugs) was not available or was lost during recording. Third, the information regarding the cause-specific death was not available in this study. Future studies are needed to determine the association between BMI and cause-specific mortality in TB patients. Notwithstanding, our study’s strength is that all eligible patients with TB were included in this analysis, so sample size was not determined according to considerations of statistical power.

This study found high mortality in patients with TB in Taipei, Taiwan, in 2011–2012. After controlling for other covariates, underweight BMI significantly increased the risk of all-cause mortality during TB treatment, but only within the first 8 weeks of treatment. Our study suggests that underweight patients should be treated carefully to reduce all-cause mortality, particularly within 8 weeks after the start of TB treatment.
